# An analysis of abstracts presented to the College on Problems of Drug Dependence meeting and subsequent publication in peer review journals

**DOI:** 10.1186/1747-597X-4-19

**Published:** 2009-11-04

**Authors:** Juan Carlos Valderrama-Zurián, Máxima Bolaños-Pizarro, Francisco Jesús Bueno-Cañigral, F Javier Álvarez, José Antonio Ontalba-Ruipérez, Rafael Aleixandre-Benavent

**Affiliations:** 1 Instituto de Historia de la Ciencia y Documentación López Piñero, Universitat de Valencia-CSIC, Valencia, Spain; 2 Plan Municipal de Drogodependencias, Concejalia de Sanidad, Ayuntamiento de Valencia, Valencia, Spain; 3 Instituto de Estudios de Alcohol y Drogas, Facultad de Medicina, Universidad de Valladolid, Valladolid, Spain; 4 Departamento de Comunicación Audiovisual, Documentación e H^a ^del Arte, Universidad Politécnica de Valencia, Valencia, Spain

## Abstract

**Background:**

Subsequent publication rate of abstracts presented at meetings is seen as an indicator of the interest and quality of the meeting. We have analyzed characteristics and rate publication in peer-reviewed journals derived from oral communications and posters presented at the 1999 College on Problems of Drug Dependence (CPDD) meeting.

**Methods:**

All 689 abstracts presented at the 1999 CPDD meeting were reviewed. In order to find the existence of publications derived from abstracts presented at that meeting, a set of bibliographical searches in the database Medline was developed in July 2006. Information was gathered concerning the abstracts, articles and journals in which they were published.

**Results:**

254 out of 689 abstracts (36.9%) gave rise to at least one publication. The oral communications had a greater likelihood of being published than did the posters (OR = 2.53, 95% CI 1.80-3.55). The average time lapse to publication of an article was 672.97 days. The number of authors per work in the subsequent publications was 4.55. The articles were published in a total of 84 journals, of which eight were indexed with the subject term Substance-Related Disorders. Psychopharmacology (37 articles, 14.5%) was the journal that published the greatest number of articles subsequent to the abstracts presented at the 1999 CPDD meeting.

**Conclusion:**

One out of every three abstracts presented to the 1999 CPDD meeting were later published in peer-reviewed journals indexed in Medline. The subsequent publication of the abstracts presented in the CPDD meetings should be actively encouraged, as this maximizes the dissemination of the scientific research and therefore the investment.

## Introduction

In the last few decades the field of knowledge of substance abuse has undergone a consolidation characterized by its ample dynamism and constant updating [1].

The abstracts (oral communications and posters) presented in the meetings provide an opportunity for professionals and researchers of the various geographical areas or countries to share information in the field of substance abuse [2].

In most scientific fields, preliminary results are often first presented at national and international meetings in the form of abstracts (oral or poster presentations). However, not all the abstracts presented in meetings are published later in article form in peer-review journals, so their adequate diffusion and quality is not guaranteed [3].

In the field of substance abuse, the congresses organized since 1938 by The College on Problems of Drug Dependence (CPDD), previously the Committee on Problems of Drug Dependence, have provided the forum in which researchers from diverse disciplines present the results of their scientific activity. The Annual Meeting Programs & Abstracts allow the abstracts of the oral communications and the posters that have been presented annually in the congresses of the CPDD since 1999 to be accessed [4]. In some of those years, the abstracts were published in *Drug and Alcohol Dependence*. As in many other scientific fields, a large percentage of the papers presented at the CPDD annual meeting do not achieve publication in a peer review journal.

The aim of the study was to analyze the publication, the characteristics of the publications and those factors influencing subsequent publication in peer-reviewed journals derived from oral communications and posters presented at the 68th meeting of the CPDD, held in 1999 in Acapulco (Mexico).

## Methods

### Data collection

In the 68th CPDD meeting, 689 abstracts (194, 28.2%, oral communications and 495, 71.8%, posters) were presented [4]. In order to find the existence of publications derived from abstracts presented at this meeting, a set of bibliographical searches in the database *Medline *[5] was developed and performed without restrictions of language or publication format, and limiting the results to the period 1999 - May 2006. The search was carried out up to 2006 in order to give a sufficient time-frame for the studies to be published.

Medline is the world's most comprehensive source of life sciences and biomedical bibliographic information. In North America, physicians typically use Medline as their sole electronic database [6].

The Medline search (carried out in July 2006) was performed using the first author's surname and initial(s). Possible different spellings of the authors' names were taken into account, because there are sometimes first and second names or two surnames, and changes may occur in the order of the surnames or the order of the names. If no subsequent article of an oral communication or poster was located, then other co-authors or a combination of the keywords from the title were searched for. The concordance between information contained in the abstract of the article and the abstract presented at the CPDD meeting was verified, thus avoiding their consideration as articles derived from works carried out by the same research team and in which different aspects of the same study were presented. It was considered that an article was a subsequent publication of an abstract presented at the 1999 CPDD meeting if the title and authors from the abstract and subsequent paper were the same, or the following three criteria were meet: i) the aims of the study were the same, ii) the number of animals, patients or sample size, when appropriate, were the same, and iii) the results - conclusions were the same.

### Main outcome measures

The following information was extracted from each of the presentations to the meeting: type of presentation (poster or oral communication), average time between abstract presentation and publication (the initial date was considered to be the day the communication or poster was presented in the congress: i.e. between 12^th ^and 17^th ^June, while the date of publication was that which appears in the Medline database in the PD (Publication Date) field and if only the month or season of publication from the journal's web page is available, the first day of that month or season was taken to be the date of publication), the number of authors per abstracts, the country of the institutions the authors belong to and whether the study was carried out on humans or not. The information extracted from the articles included: title, number of authors, publishing journal, subject area of the journal in the Journal Database of Pubmed, country of publication, document type (original research article, review, case report, letter to the editor), language, the impact factor of each article which corresponds to that of the journal in the year of the article's publication according to the Journal Citation Report^® ^[7], type of study (Basic science, clinical trials, observational study), and subject area of the journal in the Journal Database of Pubmed.

### Statistical analysis

Results were expressed as percentage and mean ± standard deviation (SD). SPSS 14.0 was used. The statistical analysis included the chi-square test, and t-test when appropriate. The Odds Ratio (OR) for likelihood of being subsequently published as an article for poster or oral communication, with 95% confidence intervals (95% CI), was calculated by using binary logistic regression analysis. A value of p ≤ 0.05 was considered to be statistically significant.

## Results

Of the 689 abstracts presented, 254 (36.9%) gave rise to at least one publication indexed in the *Medline *database. Of these 254 presentations, 102 were oral communications and 152 were posters. This means that oral communications have a greater likelihood of being published (OR = 2.53; 95% CI 1.80-3.55) than posters, since 53.1% of the total number of oral communications (n = 194) and 30.7% of the presentations in poster form (n = 495) were published at a later date in a peer-reviewed journal.

These 254 abstracts originated 255 articles in indexed journals, as 1 abstract resulted in 2 different publications. Of the 255 articles, all were original research articles, 2 were reviews and 1 a case report.

The communications were presented by institutions from 20 countries. Most (633, 91.9%) were from The U.S.A., 12 of which were collaborating with institutions from other countries (The U.K. (2), France (2), Australia (2), Puerto Rico (1), Mexico (1), Spain (1), Czech Republic (1), Russia (1) and Argentina (1)). The countries that presented the most communications without collaboration with The U.S.A. were Australia (7), Mexico and Japan (6 each), The U.K. (5) and Canada, China, Austria, Spain and Puerto Rico (4 each).

**Figure 1:**
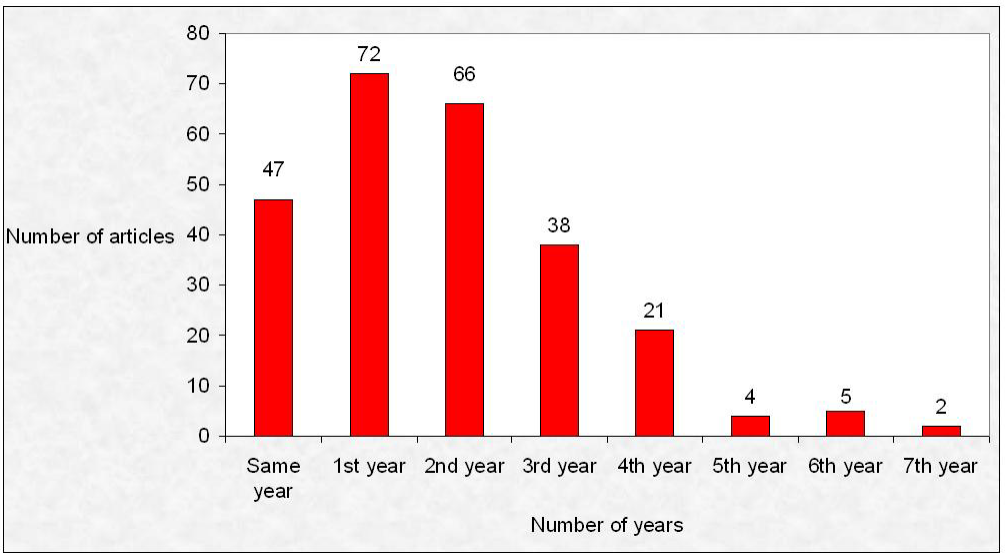
**Lapse between the presentation of a communication and its publication as an article**.

The average time lapse between the presentation of a communication and its publication as an article was of 672.97 ± 518.34 days (median = 598 days). No differences were observed with respect to the type of abstract presentation (673.54 ± 555.96, median = 596, days for oral communications and 672.58 ± 493.10, median = 598, days for posters; t = 0.015, df 253, p = 0.99). Of the total number of subsequent publications, 18.5% were published within the same year, 28.3% during the year following the celebration of the congress and 26% in the year following that (Figure 1).

The number of authors of the abstracts presented to the CPDD (n = 689) was 4.20 ± 1.95 (median = 4.0). There was no difference between the mean of the authors of the abstracts that did not give rise to a publication (n = 435, 4.18 ± 1.97, median = 4.0) and those that did (n = 254, 4.23 ± 1.93; median = 4.0, t = 0.318, df = 687, p = 0.75).

The mean number of authors in the articles published in journals (n = 255, 4.55 ± 2.16; median = 4) was greater than the number of authors who appeared in the abstracts presented to the CPDD and which resulted in a publication (t = 3.56, df = 254, p = 0.001).

No differences were found (t = 0.65, df = 253, p = 0.52) with respect to the type of abstract presentation (4.66 ± 2.38, median = 4.0, authors for articles originating in oral communications and 4.48 ± 2.00, median = 4.0, authors for those originating in posters).

The percentage of abstracts presented at the 1999 CPDD meeting that referred to studies carried out on humans was 69.7%, while in the articles subsequently published this was 62.6%. These studies (754.87 ± 538.92 days; median = 687) took longer to be published than those that were not carried out with humans (535.03 ± 451.74 days, median = 456; t = 3.49, df = 225, p = 0.001).

As for the type of study, 113 of the 255 were basic science Publications, while 100 were clinical trials (in 33 cases the descriptor was Randomized Controlled Trials), and the remaining 42 articles dealt with observational studies.

The articles were published in 84 journals indexed in *Medline *using 97 different subject areas according to the subject area of the journal in the *Journal Database of Pubmed*, the most frequent being: Substance-Related Disorders (n = 8 journals), Pharmacology (n = 8 journals), Psychopharmacology (n = 7 journals) and Psychiatry (n = 7 journals). The journal that published the greatest number of articles was *Psychopharmacology *with 37 articles (14.51%, table 1), followed by *Drug and Alcohol Dependence *with 27 (10.59%). The majority were published in U.S. (n = 54) and British (n = 19) journals. Of the 255 articles, only 8 where published in journals not indexed in the *Journal Citation Report*^® ^[7]. The mean impact factor (1999-2005) of the journals where these articles were published was 2.67 ± 1.79, (median = 2.49); there being no differences (t = 1.49, df = 250.67, p = 0.14) between the studies carried out with humans (2.86 ± 1.12, median = 2.8) and those without (2.56 ± 2.09, median = 2.21) (Table 1).

**Table 1:** Characteristics of the peer-reviewed journals indexed in Medline that have published five or more articles based on the abstracts presented in the 1999 CPDD meeting

**Peer-reviewed journal**	**Number of Published articles**	**Country**	**Subject area**	Mean impact factor, 1999-2005*
Psychopharmacology	37	Germany	Psychopharmacology	3.24

Drug and Alcohol Dependence	27	Switzerland	Alcoholism; Substance-Related Disorders	2.61

Journal of Pharmacology and Experimental Therapeutics	16	United States	Drug Therapy; Pharmacology	3.87

Pharmacology Biochemistry and Behavior	14	United States	Behavior/drug effects; Biochemistry; Pharmacology	1.96

Addiction	7	United Kingdom	Substance-Related Disorders; Tobacco Use Disorder	2.80

Addictive Behaviors	7	United Kingdom	Alcoholism; Behavior; Feeding Behavior; Obesity; Smoking; Substance-Related Disorders	1.26

American Journal on Addictions	7	United States	Behavior, Addictive; Substance-Related Disorders	1.37

Experimental and Clinical Psychopharmacology	7	United States	Psychopharmacology	1.66

American Journal of Psychiatry	6	United States	Psychiatry	7.05

Journal of Addictive Diseases	6	United States	Substance-Related Disorders	1.18

Behavioural Pharmacology	5	United Kingdom	Behavior/drug effects; Nervous System/drug effects	2.18

Brain Research	5	Netherlands	Brain; Neurology	2.41

European Journal of Pharmacology	5	Netherlands	Pharmacology	2.29

Journal of Substance Abuse Treatment	5	United States	Substance-Related Disorders	0.70

*Source: Journal Citation Report^® ^[7]

The impact factor of the articles that came from oral communications (3.04 ± 2.09, median = 2.63) was greater than that for those coming from posters (2.42 ± 1.52, median = 2.34), (t = 2.56, df = 173.24, p = 0.01).

## Discussion

Attendance at scientific congresses is usually greatly appreciated by researchers, as it allows them to keep up to date in their area of knowledge or speciality and gives them an ideal framework for establishing the necessary contacts to promote and set up collaborations [8].

The percentage of subsequent publications from the abstracts presented to the 1999 CPDD meeting (36.9%) is similar to that found in a systematic review, showing that only one-third of the abstracts presented in biomedical meetings were published in medical journals [3]. This percentage is a bit lower than the percentage found in Scherer's study across many different biomedical fields (44.5%) [9]. In a recent study by Vecchi et al. [10] based on abstract presented to CPDD meetings between 1993 to 2002, and looking specifically at randomized controlled trials and controlled clinical trials, 62% of these abstracts were published in medical journals.

Oral communications presented at the CPDD meeting were more likely to be subsequently published in peer-reviewed journals than the posters, as has been observed in Radiology [11]. This difference could be due to the fact that abstracts describing study results that are closer to completion, or completed, tend to be selected by the program committee for an oral presentation while more preliminary researches are selected for a poster.

The average time elapsed between abstract presentation at the 1999 CPDD Meeting and publication was 672.97 days, with a median of 598 days (19.9 months) similar to that observed in the area of Clinical Pharmacology and Pharmacoepidemiology (18 and 20 months, respectively) [12,13]. Almost one third of the communications (72.55%) were published within the 24 months following the celebration of the congress, a higher percentage than that found in the work of Autorino et al[14] and Dhaliwal et al [15] (with 58.8% and 15.6% respectively).

The time elapsed until publication of the study has no connection with the type of abstract presentation, as reported in the area of Paediatrics [16]. However, it is related to the type of population studied, studies not carried out with humans being published earlier. It is our belief that this may be due to the fact that studies carried out with humans more often refer to preliminary results and/or partial samples when being presented at congresses, and also to the fact that the data collection process for human studies takes longer, especially for treatment-related studies.

The average number of authors per published article is greater (0.35) than the average per abstract presented at the CPDD congress. This could be due to the fact that the teams need a greater number of professionals to write a scientific article or that new professionals join the research teams.

The results obtained show a greater tendency towards collective authorships and, in some cases, for authors of different nationalities or from different centres. This fact can be fairly positive in the following aspects: reflecting collaboration between authors, the strengthening of work groups and the increased scientific communication, all fundamental elements for scientific development [17].

The wide range of journals publishing abstracts presented in congresses has already been observed in other areas, such as pharmacoepidemiology [13]. This could probably be explained because research into substance abuse is multidisciplinary and is related to such disciplines as Neurology, Neurosciences, Psychopharmacology, Pharmacology, Psychiatry, Internal Medicine and Public Health.

*Psychopharmacology *was the journal that published the greatest number of articles, followed by *Drug and Alcohol Dependence*, the "parent" journal from the CPDD society. We are not able to provide an explanation of why these journals were selected by authors, but some aspects, such as their multidisciplinary approach, international audience and high impact factor, among other aspects, could be several of the reasons. Similar results were found in Vecchi et al. [10] study, in which CPDD meetings abstracts based on clinical trials were published mainly in Drug and Alcohol Dependence, Journal of Consulting and Clinical Psychology, and Psychopharmacology.

The impact factor is an important issue for this study due to the close relationship between the impact and the repercussions of the work, and even its quality. The journals with the greatest impact factor are also considered to be those of the greatest quality. The greater the number of communications subsequently published as articles in journals with high impact factors, the greater the quality of the scientific content of the congress will be.

The mean impact factor of the journals that published the articles between 1999-2005 was 2.82, a value greater than that of journals included in the subject category of Substance Abuse in the Journal Citation Report over the period under study (1.24), and greater than that found in publications following Paediatrics congresses [16]. Nevertheless, it is similar to the mean of Gastroenterology (2.9) [18] and lower than that of Pharmacology (3.0) [12] and Vascular (3.5) [19]. The fact that the mean value of the impact factor of the journals publishing the CPDD Meeting presentations is greater than that of the journals covering the area of Substance Abuse themselves is because some presentations were published in Psychiatry (*Archives of General Psychiatry*, 11.53) and Internal Medicine *(Annals of Internal Medicine*, 11.61) journals with high values.

As for the country of publication of the journals, it has been found that they are predominantly Anglo-American, which may be because most professionals attending the CPDD Meeting belong to that geographical area and because the *Medline *database includes mainly English language journals.

This study has some limitations that should be taken into account when interpreting the results. It examines only the 1999 CPDD meeting, so any trends could not be identified and it is unknown whether this represents a typical year or whether it is unusual. A further limitation is that the search was only carried out in *Medline*. This could mean that relevant studies may not be retrieved because that database does not include all the articles published in journals included in the Substance Abuse category of the *Journal Citation Report*^® ^(for instance, from the *Journal of Drug Issues *only 17 articles are included), or these articles are published in non-English language journals not covered by *Medline*. However, *Medline *is one of the most important electronic databases in Health Sciences, so just like other authors, we believe that few publications have been missed [13], but the possibility of underestimation of publication rate because the limiting of the search to a single database could not be discharged. Like in previous studies [16,19] we used the same criteria to perform only a Medline search when assessing the subsequent publication of the papers presented in Meetings. Another possible limitation is that changes may have occurred in the title of the articles or in the research teams that have resulted in the subsequent publication of a work being unrecognizable.

The subsequent publication of the abstracts presented in the CPDD meetings should be actively encouraged, because publication in journals is the culmination of scientific effort [20]. It is the most important way in which scientific information is disseminated, both quantitatively and qualitatively [21,22]. It is also the way junior and senior researchers have to attain recognition [23] so as to gain promotion in their professional careers and allows the economic funding dedicated to research to be used most profitably.

Actually, we can think of at least a couple reasons. One is that many of these studies have been sponsored by funding agencies or internal funds from Universities. Maximizing the dissemination of the results would help to maximize the value of the money spent. Thus, from the funder's perspective, not providing a maximum level of dissemination through publication detracts from their investment. Another reason has to do with promoting junior authors who often use presentations as a stepping stone in their career. Not publishing a publishable manuscript is to their detriment (and the detriment of their graduating University).

Overall, the publication patterns of the abstracts presented at the 1999 CPDD meeting do not differ from those observed in the Congresses of other areas of health sciences, as one in three communications presented at the 1999 CPDD meeting were published in peer-reviewed journals indexed in *Medline*.

## Competing interests

The authors declare that they have no competing interests.

## Authors' contributions

JCV and RA conceived of the study, and participated in its design and coordination. MB participed in data collection and statistical analysis. FJB and FJA participated in statistical analysis. JAO participated in the coordination. All authors read and approved the final manuscript.
